# Preparation of Multi-Motive Grid Questionnaire for Social Networking Sites Use

**DOI:** 10.1371/journal.pone.0233205

**Published:** 2020-05-21

**Authors:** Juan Hou, Yingge Zhu, Jie Tong, Yamikani Ndasauka, Xiaoyi Fang

**Affiliations:** 1 Department of Philosophy, Anhui University, Hefei, Anhui, China; 2 Department of Philosophy, Chancellor College, University of Malawi, Zomba, Malawi; 3 Institute of Developmental Psychology, Beijing Normal University, Beijing, China; Southwest University, CHINA

## Abstract

This study draws on previous studies to develop the Social Networking Sites Use Multi-Motive Grid Questionnaire (SNSU-MMG) and test its reliability and validity. The results show that social networking sites use motivation includes four factors: cognitive motivation, emotional motivation, leisure motivation and herding motivation. Confirmatory factor analysis, reliability and validity tests show that the questionnaire has good structural validity, internal consistency and split-half reliability. The SNSU-MMG can be used as a measurement tool for users' social networking sites use implicit motivation.

## 1 Introduction

### 1.1 Social Networking Sites (SNS)

According to the 44^st^ "Statistical Report on China's Internet Development Status" released in 2019, as of July 2019, the number of Internet users in China had reached 8.54 million. Research from Global Web Index indicates global Internet users spend 60 percent more time on social media over the past seven years, China spent an average of 139 minutes a day on social media in 2019, an increase of 19 minutes over 2018.SNS have become increasingly common in recent years. SNS have been integrated into people's daily lives and have a significant impact on people’s behaviors.

SNS are defined as web-based services that allow individuals to construct a public or semi-public profile and share connections with a certain list of other users [1]. The rapid development of SNS has stirred widespread concern in society. In recent years, more and more researchers are beginning to explore the motivation and usage behavior of SNS users, and how SNS attract specific groups. The main foreign SNS are Facebook, LinkedIn, MySpace, Twitter, Instagram, and others. Domestic researches on SNS focus on Weibo, WeChat, Renren and Zhihu [2–4].

### 1.2 Social networking sites use motivation

In recent years, Weibo has launched functions such as shopping, payment and entertainment. WeChat has also integrated various commercial, entertainment and lifestyle service functions. Many other SNS are also moving towards the “hypermedia” ecosystem [5].

Many researchers have done an in-depth study on seeking the main motivations to use SNS. Several studies on WeChat found that the main motivations for college students to use WeChat are seeking information and entertainment [6,7]. Emotional motivation is another important motivation driving people to use SNS [8]. On the one hand, SNS can meet some of the emotional needs of users. It not only provides possibility for users to display a positive self-image, but also provides space for users to express themselves and seek audience opportunities [9]. Those who are not good at self-expression in real life, express themselves online through SNS such as Facebook [10,11]. Second, SNS can meet the third level of Maslow's hierarchy of needs- the need for belongingness and love. In a recent survey of registered Facebook users, researchers found a positive correlation between the sense of belonging and the number of friends on SNS, that is, the more friends, the stronger the sense of belonging [12]. At the same time, SNSs not only provide users with a more convenient access to a sense of belonging, but also make up for the lack of a sense of belonging in real life. Some individuals who are marginalized in real life will log in to Facebook earlier to increase their sense of belonging and reduce pain. Individuals with high levels of loneliness and anxiety are also more likely to communicate with others through Facebook [13]. In addition, reducing boredom is one of the most important emotional motivations to use SNS [14]. SNS such as Facebook can enrich their free time by providing users with information about different contents such as games, music and news. Reducing boredom is not the only or core factor driving people to use SNS [15]. However, boredom still plays an important role in the use of SNS, and the boredom of scattered free time is still one of the important factors in the use of social networks [16].

In addition, some researchers have mentioned a herd motivation, which is characterized by following trends and being consistent with friends [17]. Studies have found the important reason why students use Facebook is that they want to make themselves look cooler and more popular [18]. Utz, Tanis and Vermeulen [19] compared the predictive power of personality and motivation variables such as popularity (need to be popular), self-esteem, attribution, vanity and other personality and motivation variables on SNS use behaviors. The results show that, for students, popular demand is the most powerful predictor of social networking usage compared to other factors.

Some Researches on users’ motivation of SNS is based on the technology acceptance model, expectation-conformation and the information system success model [20–22]. However, most of the researches are based on Katz's theory of uses and gratifications [23]. The uses and gratifications theory mean that by taking the stand of the addressees and analyzing their motivation for using media and the satisfaction of their requirements, we can investigate the effects of mass media upon people’s psychology and behavior. By encapsulating relevant researches, this theory maintains that the addressees restrain the process of media communication by means of active use of media and stresses that people's use of media is entirely based on their personal requirements and aspirations. This theory emphasizes the functions of the audience and highlights their status [24].

The uses and gratifications theory divides the needs of the mass networking sites into five categories: cognitive needs, such as access to information, knowledge and understanding; emotional needs, such as emotional, pleasant or aesthetic experiences; individual integrated needs, such as strengthening confidence and consolidating status; comprehensive social needs, such as strengthening contact with family members, and friends; and releasing stress demands, such as escaping or diverting attention [25]. Therefore, this study combines the dimensions of motivational classification with other relevant research in the basis of Katz's uses and gratifications of the five requirements of the theory.

### 1.3 Measurement of social networking sites use motivation

Currently, measuring SNS use motivation is limited to using self-assessment questionnaire to measure explicit motivation. In addition, there are few tools that are used to measure implicit motivation. Although the questionnaire technique has many advantages, some criticisms warrant consideration. For instance, Shweder and D’Andrade [26] have argued that the semantic evaluation based on memory does not measure the real behaviors. In addition, some researchers also found that the self-report of participants tend to display “social approval” and it is difficult to provide accurate information [27]. Rosenthal and Rosnow [28] found that respondents tend to behave politely and respectfully, and tend to speculate on the investigator's intentions and make "pander" responses.

In order to make up for the deficiency of explicit measurements, researchers began paying attention to implicit measurements. Wilson et al. [29] proposed the dual attitude theory, which holds that individuals can have both implicit and explicit attitudes and have different processing mechanisms. In terms of motivation, implicit motivation and explicit motivation are two independent systems [30], and many studies have shown that they are not statistically significantly correlated [31,32].

In 1976, Schmalt developed a grid technique that combines the Thematic Aptitude Test (TAT) and questionnaire measurement methods [33]. Similar to the TAT, this method uses a series of fuzzy images to arouse the motivation of the participants. The difference is that it uses a method of deciding on a series of statements to substitute for a method of writing a story in a subject's omnibus test. Fuzzy pictures (I) of different scenes and statements (J) form a matrix with i × j elements, or grid; this measurement technique is called grid technology. Compared with the complex scoring method of TAT, the test only needs to calculate the number of statements selected related to the measured motivation, which further reduces the time and energy consumption of testing, scoring and data processing.

Later, three types of grids have been developed in German thus far: the Achievement-Motive Grid [34], the Affiliation-Motive Grid [35], and the Power-Motive Grid [36]. All three grids have been proven to possess good psychometric properties with regard to factor structure, reliability, and predictive validity in a series of studies [37,38].

In 2000, Sokolowski and others combined three motivational grids to form the MMG, which can measure the three motivations with respect to its approach and avoidance—-its hopes and fears [38]. This tool improves the time-consuming and laborious disadvantage of applying three motivational grid tests separately. sentences. Instead of presenting 168 complete matrices, Items were deleted for each motivation, leaving only those with the best discriminations, reducing the response time to 15 to 20 minutes. The simplified version included only those items with the highest discriminating power. More recently, Jiao et al. [39] revised the Chinese version of the scale and proved it had good external validity.

### 1.4 Research objective

The idea of separating the implicit and explicit modes of information processing, as well as the debate on this separation, has always been a hot topic. It is ubiquitous in many areas such as cognition (such as memory), personality, attitude, self-esteem, interpersonal relationship, imprinting, self-concept, behavior and behavior control [40]. During the period 1998–2004, there were no more than 260 independent empirical studies using the IAT (implicit association test) method.

In motivational research, McClelland et al. [30] pointed out that humans have two independent motivational systems: implicit motivation and explicit motivation. The former is the unconscious and lasting emotional preference of the individual to a specific stimulus, that is, the individual's ability to experience a specific inducement as an internal reward or pleasure. The latter is the conscious attribution of one's own behavior, expressed as a goal or duty under social norms [30]. Chen [41] argues that studies on motivation and the use of SNS only involve explicit motivations that are self-aware and contained in self-concepts, and do not consider implicit motivations that are difficult for individuals to realize. However, these are associated with different aspects of personality and predict different behaviors: implicit motivation can better predict long-term spontaneous behavior and explicit motivation can predict short-term response better in specific situation. Therefore, it can be seen that the discussion of the separation of implicit and explicit motivation complies with the whole tide of psychological research.

However, the current domestic methods of measuring users' explicit motivation of users of SNS are limited to using self-assessment questionnaire. Very few methods for measuring implicit motivation are used to measure SNS use motivation. The development of measurement tools for the use of implicit motivation in SNS is essential for future research. Some researchers have used Multi-Motive Grid to measure the implicit motivation of users [42]. Therefore, this study will based on studies on the use of motivational measurements for SNS [20, 34–39] and referred to the MMG paradigm to develop a measurement tool for SNS using implicit motivation. This tool will be used to explore the structure of implicit motivation by SNS users.

## 2 Development of the social networking sites use MMG questionnaire (SNSU-MMG)

### 2.1 Development of the item pool

**Fig 1:**
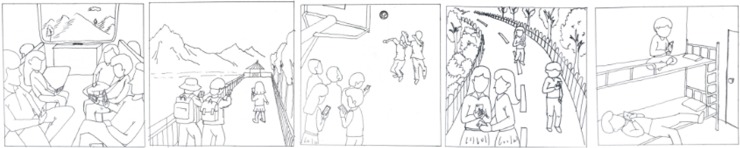
SNSU-MMG images sample.

The number of Internet users using mobile phones accounts for 97.5% of the total number of Internet users. It can be seen that the mobile phone with its portable, flexible, easy-to-get characteristics, the use of the population continues to expand. Therefore, this study chose the use of SNS via mobile phones as the main picture stimulus and the use of SNS via computers as the secondary stimulus, so as to arouse the motivation of the subjects to use SNS. Referring to the MMG paradigm of the three major motivations of previous studies, 15 fuzzy images were drawn. Each picture is a simple line of strokes containing at least two people using a mobile phone or a computer, assuming that they are using SNS such as WeChat, Weibo or Renren. When drawing, we balanced the gender of the characters in the picture, and covered a wide range of situations where the college students appear frequently. Since Sokolowski used 14 images in the MMG experiment to measure the three major motivations, this experiment deleted a picture of repeated scenes according to previous studies (such as: waiting for public transport and subway, and finally retaining only the scene of equal public transport). The remaining 14 images were scrambled to form a formal questionnaire (see Fig 1).

**Fig 2:**
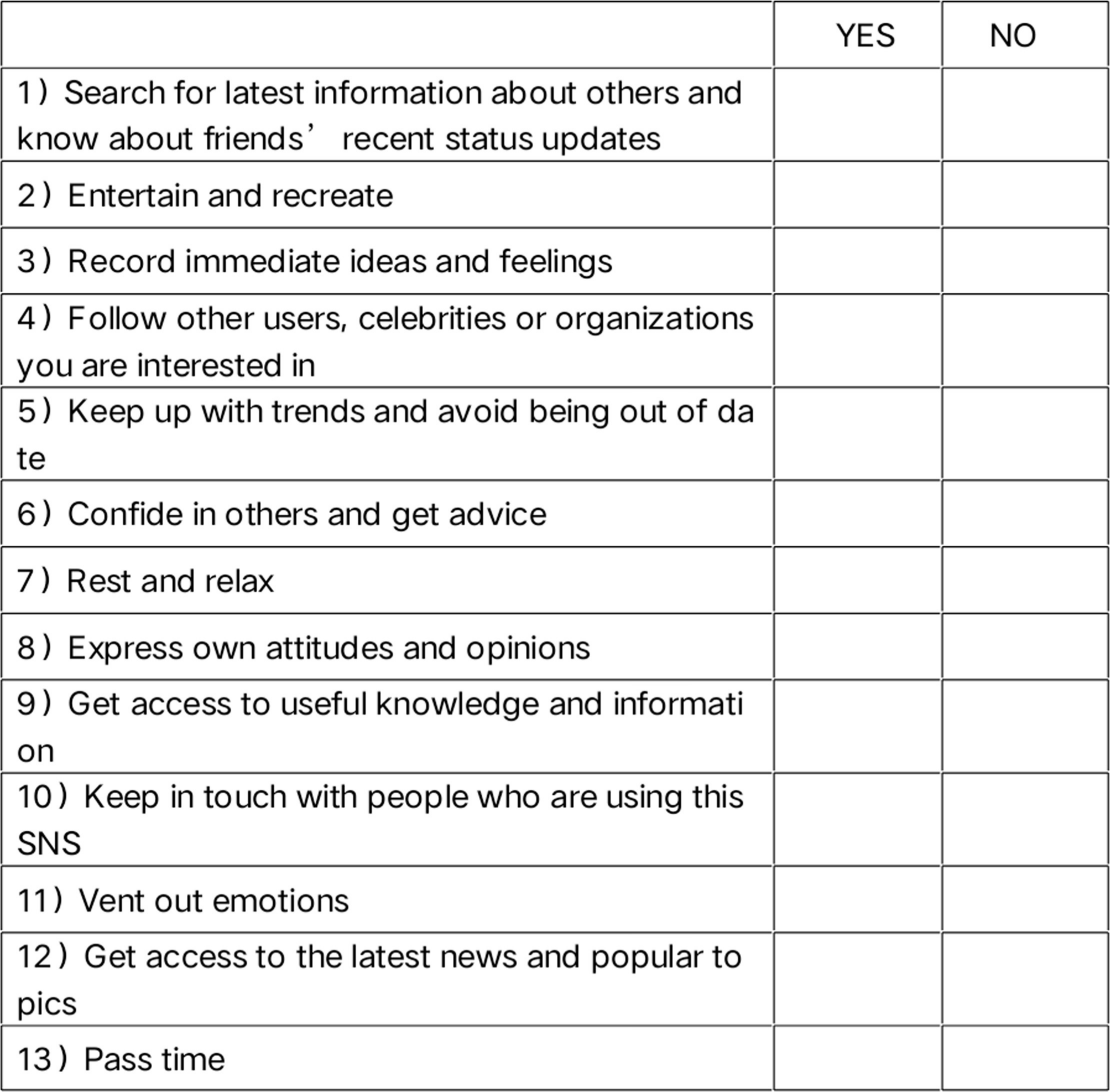
The SNSU-MMG sentences sample.

The choice of the SNSU-MMG sentences refers to the five needs proposed by the theory of use and satisfaction: cognitive needs, emotional needs, personal comprehensive needs, social comprehensive needs and entertainment needs [25]. Combined with some existing explicit motivation measurement questionnaires [43,44], 22 sentences were summarized (see Fig 2). A total of 330 units were formed at 15 × 22. Participants were asked to imagine themselves as one of the characters in the picture and make a "yes" or "no" choice.

### 2.2 Exploratory factor analysis of the SNSU-MMG

#### 2.2.1 Methods

For college students nationwide, the participants were recruited offline, 253 questionnaires were distributed, and 203 valid questionnaires were collected, including 68 males, 135 females, 197 undergraduates and 6 graduate students. The age range of the participants was 17–28 years, of which 1 person was 17 years old (*M* = 21.11, *SD* = 1.68).

The sample of this survey (*n* = 203) was used for exploratory factor analysis of the SNSU-MMG.

#### 2.2.2 Ethics statement

The study was approved by the Human Research Ethics Committee of Anhui University. All participants gave consent to participate in the study and principles expressed in the Declaration of Helsinki were closely followed. Participants were undergraduate students. Informed consent was obtained in written form from all participants.

Only one participant was 17 years old. We did not obtain consent from his guardians. This young college student was considered to have comparable intelligence and ability to adult students, and able to take charge of his behavior. According to the General principles of the Civil Law of the People's Republic of China; “A minor aged 10 or over shall be a person with limited capacity for civil conduct and may engage in civil activities appropriate to his age and intellect; in other civic activities, he shall be represented by his agent ad litem or participate with the consent of his agent ad litem” (Article 12, Chapter II). Therefore, we obtained the same consent from this participant as those above 18 years old, which was also approved by the Human Research Ethics Committee of Anhui University.

#### 2.2.3 Results

First, we ranked the participants in descending order of the total score of the questionnaire. The first 27% of the subjects were recorded as high group, and the least 27% were recorded as low group. Then, an independent sample t-test was performed on the scores of the two groups of subjects on each item. The results showed that the scores of the high and low scores on all items reached a significant level (*p*<0.01).

Second, we performed exploratory factor analysis on the initial data (n = 203). Bartlett spherical test and KMO measurement found that the Bartlett spherical test value was 3635.255 and the KMO measure was 0.929 (*p*<0.01). This meant that the data was suitable for exploratory factors analysis. Subsequently, principal component analysis and orthogonal rotation were used. According to the gravel map, determine the extraction of 4 factors.

Thirdly, the exploratory factor analysis was carried out on 22 sentences, and the items with load of 0.45 or more in two or more factors were excluded. A total of 9 items were deleted, and the remaining 13 items and 14 pictures formed the initial SNSU-MMG, and the cumulative variance contribution rate was 72.158% (see Table 1).

**Table 1:** Factor load value, eigenvalue and variance contribution rate of 15 items of the SNSU MMG.

	Factor 1		Factor 2		Factor 3		Factor 4	
	item	factor	item	factor	item	factor	item	factor
	18	0.804	3	0.798	14	0.822	10	0.864
	19	0.782	1	0.702	15	0.797	9	0.82
	21	0.75	4	0.664	13	0.741		
	20	0.732	2	0.636				
**Characteristic root**	5.125		4.792		3.229		2.728	
**Contribution rate**	23.296		21.783		14.679		12.4	
**Cumulative contribution rate**	23.396		45.079		59.758		13.4	

Factor 1 included four items, pointing to the outside world to express their own opinions and feelings, venting emotions, recording moods, etc. We named the factor as “emotional motivation”. Factor 2 included four items, referring to the knowledge and information needed to obtain the dynamics of friends, news and popular topics, etc. We named the factor as “cognitive motivation”. Factor 3 included three items, referring to relaxation, passing time and entertainment. We named the factor as “leisure motivation”. Factor 4 included two items, which referred to keeping up with people around you, following trend, keeping pace with the times. We named the factor a “herd motivation”.

Fourth, we performed a reliability test that showed the internal consistency (Cronbach's α coefficient) of the 14 images was 0.938. The reliability test of the initial questionnaire showed that the score of the questionnaire was 0.857, and the internal consistency (Cronbach's α coefficient) of the four subtests of emotional motivation, cognitive motivation, leisure motivation and herd motivation were 0.899, 0.851, 0.890, 0.839 respectively.

Finally, we performed a correlation test between each picture and the total score is between 0.555 and 0.827, and both were found to be significantly correlated (*p*< 0.01). Another correlation analysis between each item and the total score showed that items significantly correlated with the total score (*p*<0.01), and the correlation coefficient was 0.465~0.838. In addition, the correlation coefficient between the questionnaire scores of the SNSU-MMG and the total score of the SNSU-MMG was between 0.651 and 0.879; and correlation coefficient reached a significant level (*p*<0.01).

## 3 Confirmatory factor analysis and correlation analysis of the SNSU-MMG

### 3.1 Measures

#### Motives for using the SNS

We employed the Motives for using the SNS [45] in our study to measure the relationship between explicit use motivation and implicit use motivation. The questionnaire has 20 statements (items).The questions were rated on a five-point, Likert type scale, with 1 being “Strongly Disagree” and 5 being “Strong Agree”.

#### SNSU-MMG

Our questionnaire comprised 13 items and 14 pictures. Participants were asked to imagine themselves as one of the characters in the picture and make a "yes" or "no" choice.

### 3.2 Methods

872 questionnaires were distributed online and offline. Online questionnaires were distributed through Questionnaire star. For the offline method, the questionnaire was distributed to the surrounding areas and student groups of a public university in China, and finally 787 valid questionnaires were collected. The age range of the participants was 17–25 years, of which 6 persons were 17 years old (*M* = 19.66, *SD* = 1.09). Among them, 382 were male and 405 were female.

The sample of this survey (*n* = 787) was used for confirmatory factor analysis and correlation analysis.

### 3.3 Result

#### 3.3.1 Confirmatory factor analysis results

**Fig 3:**
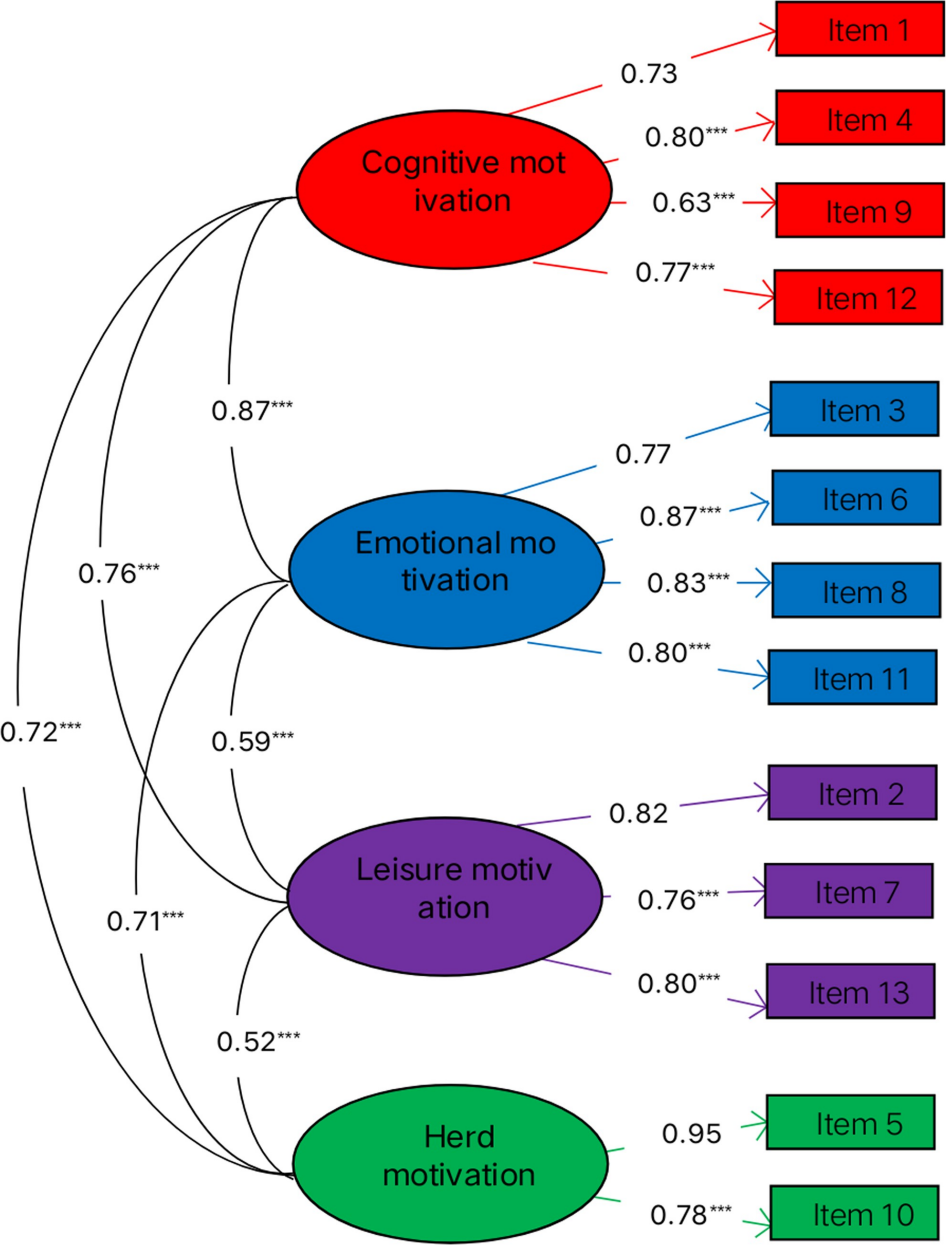
Confirmatory factor analysis of the four-factor SNSU MMG. *p<0.05; **p<0.01; *** p< 0.001, the same below.

A confirmatory factor analysis was performed on the retest data (n = 787). The results showed that the overall fit indicators and the fit indices were within a reasonable range: χ2/df = 3.89, CFI = 0.99, TLI = 0.97, AGFI = 0.94, NFI = 0.98, IFI = 0.99, RFI = 0.95, GFI = 0.98, RMSEA = 0.06. The results of the confirmatory factor analysis supported the rationality of the SNSU-MMG model initially constructed in this study. Fig 3 shows a model of a standardized estimate model.

#### 3.3.2 Correlation analysis between explicit use motivation and implicit use motivation

We performed a correlation test between the motivational questionnaire for SNS and MMG. Results showed that SNSU-MMG scored were not significantly correlated with the motivation scores of the explicit questionnaire (*p*>0.01), or significantly correlated but the correlation coefficient values were less than 0.2. The study found that below 0.2 was considered to be zero- or weakly correlated [46,47]. Therefore, significant correlation can be ignored (See Table 2).

**Table 2:** Correlations between implicit motivation and explicit motivation used by SNS (n = 787).

	Cognitive motivation	Emotional motivation	Leisure motivation	Herd motivation	Seeking friends	Seeking convenience	Seeking social support	Seeking information
Emotional motivation	.76***							
Leisure motivation	.63***	.53***						
Herd motivation	.58***	.63***	.47***					
Seeking friends	.11**	.14**	.04	.09*				
Seeking convenience	.03	-.04	.07	-.03	.39***			
Seeking social support	-.09*	.15***	.06	.11**	.46***	.43***		
Seeking information	.06	-.01	-.04	-.05	.35***	.31***	.31***	
Seeking entertainment	.03	.03	.13***	.06	.32***	.43***	.41***	.35***

## 4 Discussion

In conclusion, the SNSU-MMG has good validity and reliability, which meets the psychometric requirements and can be applied in the Chinese college students. And the motivations measured in the SNSU-MMG was minimally and insignificantly related to the motivations measured in the explicit questionnaire.

### 4.1 Psychometric properties of the SNSU-MMG

In previous studies, researchers basically used self-assessment questionnaires to measure the motivation of users of SNS. This study innovatively employed multi-motivation grid technology, which combines the theme projection test and the semi-projection method of questionnaire measurement to measure the implicit motivation of users of SNS. It is the first time that MMG has been applied to the study of more than three motivations. It has good internal consistency and structural validity.

On the basis of the previous studies, this study combined with the uses and gratifications of Katz and the questionnaire compiled by the predecessors, constructing the theoretical structure of the motivation of SNS use. Meanwhile, this study developed the MMG to measure the motivation of SNS with reference to Sokolowski's MMG. The blurred images used in this experiment were originally drawn with reference to the image of MMG. Then through exploratory factor analysis, four-factor structure of the questionnaire was preliminarily established, namely, cognitive motivation, emotional motivation, leisure motivation and herd motivation.

Through correlation analysis between various factors and total scores, results showed that the correlation coefficient between each factor and the total score is between 0.56 and 0.91, and both reach a significant level (*p*<0.01). Following the procedure of cross-validation, a confirmatory factor analysis was carried out after the formal questionnaire was re-sampled. Results showed the 4-factor structural model of the SNSU-MMG had better fit the observation data and supported results of exploratory factor analysis. At the same time, this study examined the sub-confidence of the questionnaire and the internal consistency of each factor, all of which met psychometric values.

Overall, the SNSU-MMG showed adequate psychometric properties within the study’s sample of college students from China. With the gradual integration of SNS into people's daily life, and has a significant impact on people's behavior, in the future, this instrument should be assessed using a larger and more representative sample. We envisage that relatively the same results would be obtained in a larger and more representative sample.

### 4.2 Implicit use motivation and explicit use motivation

Although the academic community has clearly distinguished the concepts of implicit and explicit motivation in the past than 20 years, the debate on the methods for direct and indirect measurement of motivation has been long-standing. In essence, motivation measures the debate between direct and indirect methods, that is, the explicit and implicit measurement methods of motivation. This study compared the correlation between implicit use motivation and explicit use motivation, and found that the motivations measured in the SNSU-MMG was minimally and insignificantly related to the motivations measured in the explicit questionnaire. This shows that the implicit use motivation and explicit use motivation of SNS are two separate systems, supporting the argument that McClelland proposed in 1989 that implicit motivation and explicit motivation are two independent systems [29].

The measurement of motivation is accompanied by the development of measurement technology, and has experienced a development process of implicit and explicit methods. Currently, with the development of implicit motivation measurement methods [48,49], people re-examine the reliability and validity of implicit motivation measurement. It is found that individual motivation is often interfered with by social expectations and presents a certain motivation through self-decoration in order to achieve the desired motivation of society [50,51]. The motivations measured by the explicit questionnaire are generally the explicit motivation of the participants, which are the motivations after finishing, and often cannot express the real motivation of the subjects. At the same time, implicit motivations seem to be more easily arousing and responding to nonverbal cues, which can better predict the non-declarative measurement of motivation and behavior [52]. These results should prove that the implicit measurement has good discriminant validity and is necessary for future research.

### 4.3 Limitations and prospects

The MMG developed by Sokolowski is based on the Achievement- Motive Grid [34], the Affiliation-Motive Grid [35], and the Power-Motive Grid [36]. Participants were asked to rate the degree to which the pictures and sentences could represent the motivations (achievement, affiliation and power), and 14 pictures with 12 statements were selected to show the three kinds of motivation. In selection of pictures, Sokolowski retained six pictures with low fuzziness, which only reflected one kind of motivation; 6 pictures with moderate fuzziness that evoked two kinds of motives; and 2 pictures with high fuzziness which could evoke three kinds of motives at the same time. [38] However, since there were no previous images for reference in this study, only fuzzy images were drawn with reference to the characteristics of MMG fuzzy images, which may need to be further improved in the future.

Since MMG is still a new method in measuring the motivation of users of SNS, there are still numerous further considerations. For example, in considering the picture scene, it is necessary to be cautious when using it to non-college students. This is because our study focused on college students, some of the scenes on campus (such as the bedroom, library, classroom, etc.). Future research can further improve and revise the MMG pictures and statements in this study to make it more externally valid and can be extended to non-college students. At present, there are few empirical studies measuring the use of implicit motivation in SNS. Most studies choose to use questionnaires to measure explicit motivation. Therefore, in the future, we can pay more attention to implicit motivation, and use the experimental methods such as IAT and MMG to compare the implicit motivation with the explicit motivation.

## Supporting information

S1 Data (DS_STORE)

S1 File Motives for using the SNS (original language). (DOCX)

S2 File Social Networking Sites Use Multi-Motive Grid Questionnaire (SNSU-MMG) (original language). (DOCX)

S3 File Motives for using the SNS. (DOCX)

S4 File Social Networking Sites Use Multi-Motive Grid Questionnaire (SNSU-MMG). (DOCX)
