# Supplementary material for: Preparation of Multi-Motive Grid Questionnaire for Social Networking Sites Use
Source: PLoS One. 2020 May 21;15(5):e0233205. doi: 10.1371/journal.pone.0233205 (PMC7241767; doi:10.1371/journal.pone.0233205)
Supplement: S1 File — (DOCX) [file pone.0233205.s002.docx]

**社交网站使用动机量表**

下面是一些关于你对社交网站使用动机的描述,请你根据自己的实际情况,仔细作答,请选择最接近自己情况的选项。

| **题目** | **完全不同意** | **不同意** | **中立** | **同意** | **非常同意** |
| --- | --- | --- | --- | --- | --- |
| 1. **认识新朋友** | 1 | 2 | 3 | 4 | 5 |
| 1. **发现与我相似的人** | 1 | 2 | 3 | 4 | 5 |
| 1. **与我兴趣相投的人聊天** | 1 | 2 | 3 | 4 | 5 |
| 1. **与我喜欢的人保持联系** | 1 | 2 | 3 | 4 | 5 |
| 1. **无时无刻不使用它** | 1 | 2 | 3 | 4 | 5 |
| 1. **使用它很方便** | 1 | 2 | 3 | 4 | 5 |
| 1. **使用它很简单** | 1 | 2 | 3 | 4 | 5 |
| 1. **以最少的代价来获得我想要的** | 1 | 2 | 3 | 4 | 5 |
| 1. **让自己的情绪轻易地对愿意倾听的人发泄出来** | 1 | 2 | 3 | 4 | 5 |
| 1. **向那些会同情我的人倾诉我的愤怒** | 1 | 2 | 3 | 4 | 5 |
| 1. **说出我的问题，并得到建议** | 1 | 2 | 3 | 4 | 5 |
| 1. **让他人知道我在乎他们的感受** | 1 | 2 | 3 | 4 | 5 |
| 1. **学习不知道的东西** | 1 | 2 | 3 | 4 | 5 |
| 1. **做研究** | 1 | 2 | 3 | 4 | 5 |
| 1. **学习有用的东西** | 1 | 2 | 3 | 4 | 5 |
| 1. **获得新观点** | 1 | 2 | 3 | 4 | 5 |
| 1. **忘记工作以及其他事情** | 1 | 2 | 3 | 4 | 5 |
| 1. **放松** | 1 | 2 | 3 | 4 | 5 |
| 1. **感到幸福** | 1 | 2 | 3 | 4 | 5 |
| 1. **打发时间** | 1 | 2 | 3 | 4 | 5 |

**我使用社交网站来:**
