# Supplementary material for: Preparation of Multi-Motive Grid Questionnaire for Social Networking Sites Use
Source: PLoS One. 2020 May 21;15(5):e0233205. doi: 10.1371/journal.pone.0233205 (PMC7241767; doi:10.1371/journal.pone.0233205)
Supplement: S2 File — (DOCX) [file pone.0233205.s003.docx]

**社交网站使用动机MMG问卷**

接下您将会看到一系列描绘每天各种场景的图片。假设下图中使用手机或电脑的人物都正在使用社交网站（如：QQ、微信、微博、人人网、Facebook、Twitter、Instagram等）。请您在答题时尽量将自己想象成下图中使用社交网站的某一个人物，发挥想象力去推测图片中发生的情境，并回答以下问题：

**“为什么在这个场合使用社交媒体？”**

图片下方，您将看到一系列句子，请判断句子是否能回答上述问题。如恰当，请选择“是”；反之，请选择“否”。您的答案无对错之分，请您根据即时的反应作出判断。


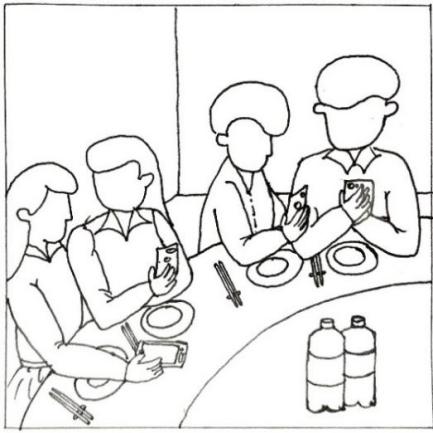


|  | 是 | 否 |
| --- | --- | --- |
| 1）搜寻他人的最新信息，了解朋友动态 |  |  |
| 2）娱乐消遣 |  |  |
| 3）记录心情和感受 |  |  |
| 4）关注感兴趣的用户、明星或组织 |  |  |
| 5）跟随潮流，不落伍 |  |  |
| 6）向他人倾诉并获得建议 |  |  |
| 7）放松和休息 |  |  |
| 8）表达自己的态度和观点 |  |  |
| 9）获取有用的知识、信息 |  |  |
| 10）身边的人都在使用，与他们保持一致 |  |  |
| 11）宣泄个人情绪 |  |  |
| 12）获得最新、最快的新闻资讯和流行话题 |  |  |
| 13）打发时间 |  |  |


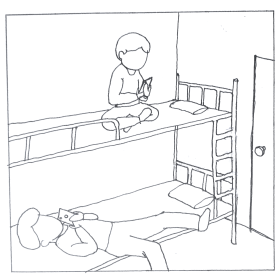


|  | 是 | 否 |
| --- | --- | --- |
| 1）搜寻他人的最新信息，了解朋友动态 |  |  |
| 2）娱乐消遣 |  |  |
| 3）记录心情和感受 |  |  |
| 4）关注感兴趣的用户、明星或组织 |  |  |
| 5）跟随潮流，不落伍 |  |  |
| 6）向他人倾诉并获得建议 |  |  |
| 7）放松和休息 |  |  |
| 8）表达自己的态度和观点 |  |  |
| 9）获取有用的知识、信息 |  |  |
| 10）身边的人都在使用，与他们保持一致 |  |  |
| 11）宣泄个人情绪 |  |  |
| 12）获得最新、最快的新闻资讯和流行话题 |  |  |
| 13）打发时间 |  |  |


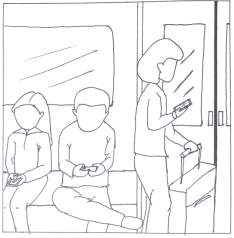


|  | 是 | 否 |
| --- | --- | --- |
| 1）搜寻他人的最新信息，了解朋友动态 |  |  |
| 2）娱乐消遣 |  |  |
| 3）记录心情和感受 |  |  |
| 4）关注感兴趣的用户、明星或组织 |  |  |
| 5）跟随潮流，不落伍 |  |  |
| 6）向他人倾诉并获得建议 |  |  |
| 7）放松和休息 |  |  |
| 8）表达自己的态度和观点 |  |  |
| 9）获取有用的知识、信息 |  |  |
| 10）身边的人都在使用，与他们保持一致 |  |  |
| 11）宣泄个人情绪 |  |  |
| 12）获得最新、最快的新闻资讯和流行话题 |  |  |
| 13）打发时间 |  |  |


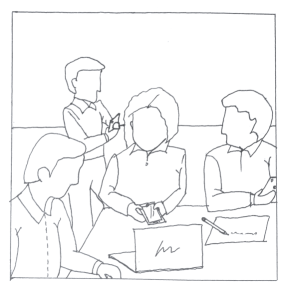


|  | 是 | 否 |
| --- | --- | --- |
| 1）搜寻他人的最新信息，了解朋友动态 |  |  |
| 2）娱乐消遣 |  |  |
| 3）记录心情和感受 |  |  |
| 4）关注感兴趣的用户、明星或组织 |  |  |
| 5）跟随潮流，不落伍 |  |  |
| 6）向他人倾诉并获得建议 |  |  |
| 7）放松和休息 |  |  |
| 8）表达自己的态度和观点 |  |  |
| 9）获取有用的知识、信息 |  |  |
| 10）身边的人都在使用，与他们保持一致 |  |  |
| 11）宣泄个人情绪 |  |  |
| 12）获得最新、最快的新闻资讯和流行话题 |  |  |
| 13）打发时间 |  |  |


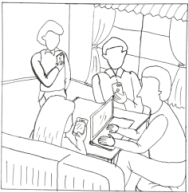


|  | 是 | 否 |
| --- | --- | --- |
| 1）搜寻他人的最新信息，了解朋友动态 |  |  |
| 2）娱乐消遣 |  |  |
| 3）记录心情和感受 |  |  |
| 4）关注感兴趣的用户、明星或组织 |  |  |
| 5）跟随潮流，不落伍 |  |  |
| 6）向他人倾诉并获得建议 |  |  |
| 7）放松和休息 |  |  |
| 8）表达自己的态度和观点 |  |  |
| 9）获取有用的知识、信息 |  |  |
| 10）身边的人都在使用，与他们保持一致 |  |  |
| 11）宣泄个人情绪 |  |  |
| 12）获得最新、最快的新闻资讯和流行话题 |  |  |
| 13）打发时间 |  |  |


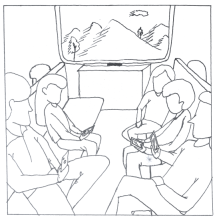


|  | 是 | 否 |
| --- | --- | --- |
| 1）搜寻他人的最新信息，了解朋友动态 |  |  |
| 2）娱乐消遣 |  |  |
| 3）记录心情和感受 |  |  |
| 4）关注感兴趣的用户、明星或组织 |  |  |
| 5）跟随潮流，不落伍 |  |  |
| 6）向他人倾诉并获得建议 |  |  |
| 7）放松和休息 |  |  |
| 8）表达自己的态度和观点 |  |  |
| 9）获取有用的知识、信息 |  |  |
| 10）身边的人都在使用，与他们保持一致 |  |  |
| 11）宣泄个人情绪 |  |  |
| 12）获得最新、最快的新闻资讯和流行话题 |  |  |
| 13）打发时间 |  |  |


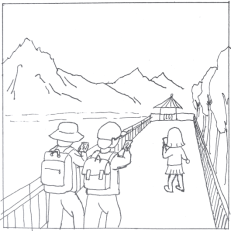


|  | 是 | 否 |
| --- | --- | --- |
| 1）搜寻他人的最新信息，了解朋友动态 |  |  |
| 2）娱乐消遣 |  |  |
| 3）记录心情和感受 |  |  |
| 4）关注感兴趣的用户、明星或组织 |  |  |
| 5）跟随潮流，不落伍 |  |  |
| 6）向他人倾诉并获得建议 |  |  |
| 7）放松和休息 |  |  |
| 8）表达自己的态度和观点 |  |  |
| 9）获取有用的知识、信息 |  |  |
| 10）身边的人都在使用，与他们保持一致 |  |  |
| 11）宣泄个人情绪 |  |  |
| 12）获得最新、最快的新闻资讯和流行话题 |  |  |
| 13）打发时间 |  |  |


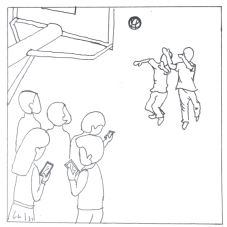


|  | 是 | 否 |
| --- | --- | --- |
| 1）搜寻他人的最新信息，了解朋友动态 |  |  |
| 2）娱乐消遣 |  |  |
| 3）记录心情和感受 |  |  |
| 4）关注感兴趣的用户、明星或组织 |  |  |
| 5）跟随潮流，不落伍 |  |  |
| 6）向他人倾诉并获得建议 |  |  |
| 7）放松和休息 |  |  |
| 8）表达自己的态度和观点 |  |  |
| 9）获取有用的知识、信息 |  |  |
| 10）身边的人都在使用，与他们保持一致 |  |  |
| 11）宣泄个人情绪 |  |  |
| 12）获得最新、最快的新闻资讯和流行话题 |  |  |
| 13）打发时间 |  |  |


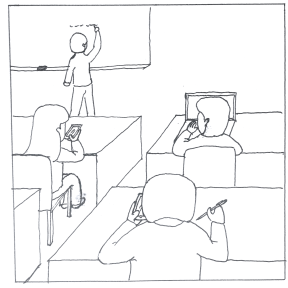


|  | 是 | 否 |
| --- | --- | --- |
| 1）搜寻他人的最新信息，了解朋友动态 |  |  |
| 2）娱乐消遣 |  |  |
| 3）记录心情和感受 |  |  |
| 4）关注感兴趣的用户、明星或组织 |  |  |
| 5）跟随潮流，不落伍 |  |  |
| 6）向他人倾诉并获得建议 |  |  |
| 7）放松和休息 |  |  |
| 8）表达自己的态度和观点 |  |  |
| 9）获取有用的知识、信息 |  |  |
| 10）身边的人都在使用，与他们保持一致 |  |  |
| 11）宣泄个人情绪 |  |  |
| 12）获得最新、最快的新闻资讯和流行话题 |  |  |
| 13）打发时间 |  |  |


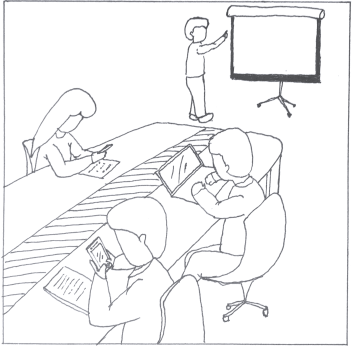


|  | 是 | 否 |
| --- | --- | --- |
| 1）搜寻他人的最新信息，了解朋友动态 |  |  |
| 2）娱乐消遣 |  |  |
| 3）记录心情和感受 |  |  |
| 4）关注感兴趣的用户、明星或组织 |  |  |
| 5）跟随潮流，不落伍 |  |  |
| 6）向他人倾诉并获得建议 |  |  |
| 7）放松和休息 |  |  |
| 8）表达自己的态度和观点 |  |  |
| 9）获取有用的知识、信息 |  |  |
| 10）身边的人都在使用，与他们保持一致 |  |  |
| 11）宣泄个人情绪 |  |  |
| 12）获得最新、最快的新闻资讯和流行话题 |  |  |
| 13）打发时间 |  |  |


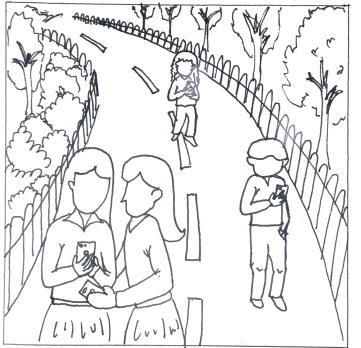


|  | 是 | 否 |
| --- | --- | --- |
| 1）搜寻他人的最新信息，了解朋友动态 |  |  |
| 2）娱乐消遣 |  |  |
| 3）记录心情和感受 |  |  |
| 4）关注感兴趣的用户、明星或组织 |  |  |
| 5）跟随潮流，不落伍 |  |  |
| 6）向他人倾诉并获得建议 |  |  |
| 7）放松和休息 |  |  |
| 8）表达自己的态度和观点 |  |  |
| 9）获取有用的知识、信息 |  |  |
| 10）身边的人都在使用，与他们保持一致 |  |  |
| 11）宣泄个人情绪 |  |  |
| 12）获得最新、最快的新闻资讯和流行话题 |  |  |
| 13）打发时间 |  |  |


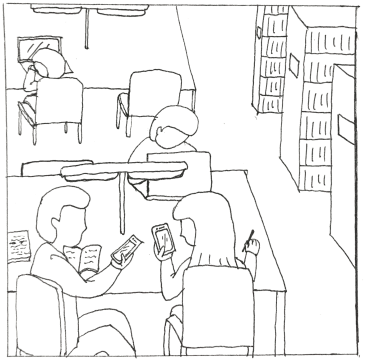


|  | 是 | 否 |
| --- | --- | --- |
| 1）搜寻他人的最新信息，了解朋友动态 |  |  |
| 2）娱乐消遣 |  |  |
| 3）记录心情和感受 |  |  |
| 4）关注感兴趣的用户、明星或组织 |  |  |
| 5）跟随潮流，不落伍 |  |  |
| 6）向他人倾诉并获得建议 |  |  |
| 7）放松和休息 |  |  |
| 8）表达自己的态度和观点 |  |  |
| 9）获取有用的知识、信息 |  |  |
| 10）身边的人都在使用，与他们保持一致 |  |  |
| 11）宣泄个人情绪 |  |  |
| 12）获得最新、最快的新闻资讯和流行话题 |  |  |
| 13）打发时间 |  |  |


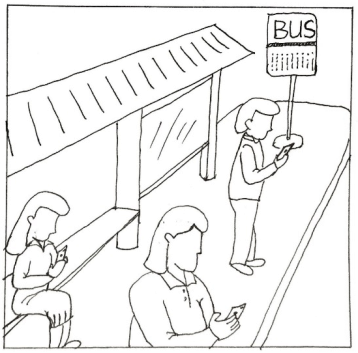


|  | 是 | 否 |
| --- | --- | --- |
| 1）搜寻他人的最新信息，了解朋友动态 |  |  |
| 2）娱乐消遣 |  |  |
| 3）记录心情和感受 |  |  |
| 4）关注感兴趣的用户、明星或组织 |  |  |
| 5）跟随潮流，不落伍 |  |  |
| 6）向他人倾诉并获得建议 |  |  |
| 7）放松和休息 |  |  |
| 8）表达自己的态度和观点 |  |  |
| 9）获取有用的知识、信息 |  |  |
| 10）身边的人都在使用，与他们保持一致 |  |  |
| 11）宣泄个人情绪 |  |  |
| 12）获得最新、最快的新闻资讯和流行话题 |  |  |
| 13）打发时间 |  |  |


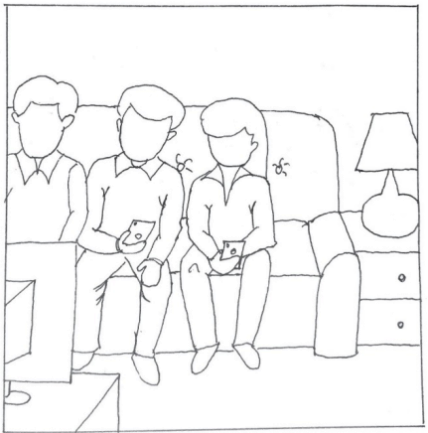


|  | 是 | 否 |
| --- | --- | --- |
| 1）搜寻他人的最新信息，了解朋友动态 |  |  |
| 2）娱乐消遣 |  |  |
| 3）记录心情和感受 |  |  |
| 4）关注感兴趣的用户、明星或组织 |  |  |
| 5）跟随潮流，不落伍 |  |  |
| 6）向他人倾诉并获得建议 |  |  |
| 7）放松和休息 |  |  |
| 8）表达自己的态度和观点 |  |  |
| 9）获取有用的知识、信息 |  |  |
| 10）身边的人都在使用，与他们保持一致 |  |  |
| 11）宣泄个人情绪 |  |  |
| 12）获得最新、最快的新闻资讯和流行话题 |  |  |
| 13）打发时间 |  |  |
