# Supplementary material for: Preparation of Multi-Motive Grid Questionnaire for Social Networking Sites Use
Source: PLoS One. 2020 May 21;15(5):e0233205. doi: 10.1371/journal.pone.0233205 (PMC7241767; doi:10.1371/journal.pone.0233205)
Supplement: S3 File — (DOCX) [file pone.0233205.s004.docx]

**Motives for using the SNS**

These are some descriptions about motives for social networking usage. Please choose the numbers that fit you best.

**I use social networking sites to …**

| **Item** | **Strongly Disagree** | **disagree** | **neutral** | **agree** | **Strong Agree** |
| --- | --- | --- | --- | --- | --- |
| **1.Meet new people.** | 1 | 2 | 3 | 4 | 5 |
| **2.Find others like me.** | 1 | 2 | 3 | 4 | 5 |
| **3.Talk with people with the same interests** | 1 | 2 | 3 | 4 | 5 |
| **4.Hang out with people I enjoy** | 1 | 2 | 3 | 4 | 5 |
| **5.Use it anytime, anywhere.** | 1 | 2 | 3 | 4 | 5 |
| **6.Use it conveniently** | 1 | 2 | 3 | 4 | 5 |
| **7.Use it easily.** | 1 | 2 | 3 | 4 | 5 |
| **8.Get what I want for less effort** | 1 | 2 | 3 | 4 | 5 |
| **9.Let out my emotions easily to others who will** | 1 | 2 | 3 | 4 | 5 |
| **10.Express my anger to others who will sympathize** | 1 | 2 | 3 | 4 | 5 |
| **11.Talk out my problems and get advice.** | 1 | 2 | 3 | 4 | 5 |
| **12.Let others know I care about their feelings.** | 1 | 2 | 3 | 4 | 5 |
| **13.Learn about unknown things.** | 1 | 2 | 3 | 4 | 5 |
| **14.Do research.** | 1 | 2 | 3 | 4 | 5 |
| **15.Learn about useful things.** | 1 | 2 | 3 | 4 | 5 |
| **16.Get new ideas.** | 1 | 2 | 3 | 4 | 5 |
| **17.Forget about work or other things.** | 1 | 2 | 3 | 4 | 5 |
| **18.Relax.** | 1 | 2 | 3 | 4 | 5 |
| **19.Feel excited.** | 1 | 2 | 3 | 4 | 5 |
| **20.Pass the time.** | 1 | 2 | 3 | 4 | 5 |
