# Supplementary material for: Preparation of Multi-Motive Grid Questionnaire for Social Networking Sites Use
Source: PLoS One. 2020 May 21;15(5):e0233205. doi: 10.1371/journal.pone.0233205 (PMC7241767; doi:10.1371/journal.pone.0233205)
Supplement: S4 File — (DOCX) [file pone.0233205.s005.docx]

**Social Networking Sites Use Multi-Motive Grid Questionnaire (SNSU-MMG)**

In the following questionnaire, you will be showed **14 images** depicting a variety of daily scenes. The images assume that *the person in the pictures below is using Social networking sites (Facebook, Twitter, Instagram etc.) on cellphones or laptops.*

Please imagine yourself as one of the individuals who is using SNS and speculate the circumstance with this question:

**Why using Social networking sites on this occasion?**

Below the picture, you will be showed a series of sentences, please determine whether the sentence can answer the question above. If appropriate, choose YES. If not, choose NO. There are no standard answers for each question and the result will just be used for our academic study. Your answers will be kept completely confidential. Please answer the questions according to your true feelings.


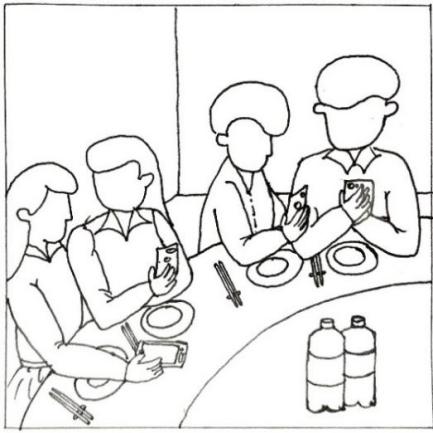


|  | YES | NO |
| --- | --- | --- |
| 1）Search for the latest information about others and know about friends’ recent status |  |  |
| 2）Entertain and recreate |  |  |
| 3）Record immediate ideas and feelings |  |  |
| 4）Follow the users, celebrities or organizations interested |  |  |
| 5）Keep up with trends and avoid being outdated |  |  |
| 6）Confide in others and get advice |  |  |
| 7）Rest and relax |  |  |
| 8）Express own attitudes and opinions |  |  |
| 9）Get access to useful knowledge and information |  |  |
| 10）Keep in line with people around who are using this SNS |  |  |
| 11）Give vent to emotions |  |  |
| 12）Get access to the latest news and popular topics |  |  |
| 13）Pass time |  |  |


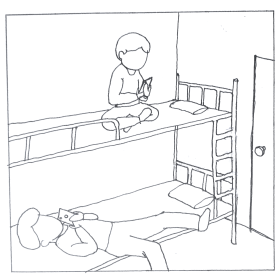


|  | YES | NO |
| --- | --- | --- |
| 1）Search for the latest information about others and know about friends’ recent status |  |  |
| 2）Entertain and recreate |  |  |
| 3）Record immediate ideas and feelings |  |  |
| 4）Follow the users, celebrities or organizations interested |  |  |
| 5）Keep up with trends and avoid being outdated |  |  |
| 6）Confide in others and get advice |  |  |
| 7）Rest and relax |  |  |
| 8）Express own attitudes and opinions |  |  |
| 9）Get access to useful knowledge and information |  |  |
| 10）Keep in line with people around who are using this SNS |  |  |
| 11）Give vent to emotions |  |  |
| 12）Get access to the latest news and popular topics |  |  |
| 13）Pass time |  |  |


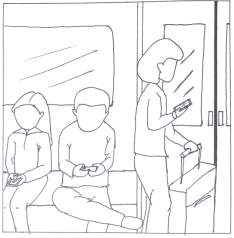


|  | YES | NO |
| --- | --- | --- |
| 1）Search for the latest information about others and know about friends’ recent status |  |  |
| 2）Entertain and recreate |  |  |
| 3）Record immediate ideas and feelings |  |  |
| 4）Follow the users, celebrities or organizations interested |  |  |
| 5）Keep up with trends and avoid being outdated |  |  |
| 6）Confide in others and get advice |  |  |
| 7）Rest and relax |  |  |
| 8）Express own attitudes and opinions |  |  |
| 9）Get access to useful knowledge and information |  |  |
| 10）Keep in line with people around who are using this SNS |  |  |
| 11）Give vent to emotions |  |  |
| 12）Get access to the latest news and popular topics |  |  |
| 13）Pass time |  |  |


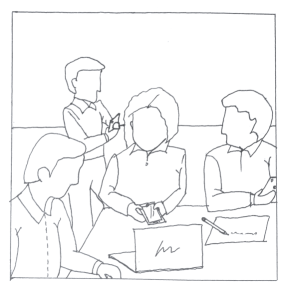


|  | YES | NO |
| --- | --- | --- |
| 1）Search for the latest information about others and know about friends’ recent status |  |  |
| 2）Entertain and recreate |  |  |
| 3）Record immediate ideas and feelings |  |  |
| 4）Follow the users, celebrities or organizations interested |  |  |
| 5）Keep up with trends and avoid being outdated |  |  |
| 6）Confide in others and get advice |  |  |
| 7）Rest and relax |  |  |
| 8）Express own attitudes and opinions |  |  |
| 9）Get access to useful knowledge and information |  |  |
| 10）Keep in line with people around who are using this SNS |  |  |
| 11）Give vent to emotions |  |  |
| 12）Get access to the latest news and popular topics |  |  |
| 13）Pass time |  |  |


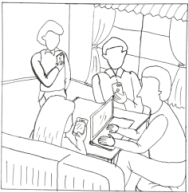


|  | YES | NO |
| --- | --- | --- |
| 1）Search for the latest information about others and know about friends’ recent status |  |  |
| 2）Entertain and recreate |  |  |
| 3）Record immediate ideas and feelings |  |  |
| 4）Follow the users, celebrities or organizations interested |  |  |
| 5）Keep up with trends and avoid being outdated |  |  |
| 6）Confide in others and get advice |  |  |
| 7）Rest and relax |  |  |
| 8）Express own attitudes and opinions |  |  |
| 9）Get access to useful knowledge and information |  |  |
| 10）Keep in line with people around who are using this SNS |  |  |
| 11）Give vent to emotions |  |  |
| 12）Get access to the latest news and popular topics |  |  |
| 13）Pass time |  |  |


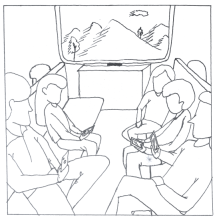


|  | YES | NO |
| --- | --- | --- |
| 1）Search for the latest information about others and know about friends’ recent status |  |  |
| 2）Entertain and recreate |  |  |
| 3）Record immediate ideas and feelings |  |  |
| 4）Follow the users, celebrities or organizations interested |  |  |
| 5）Keep up with trends and avoid being outdated |  |  |
| 6）Confide in others and get advice |  |  |
| 7）Rest and relax |  |  |
| 8）Express own attitudes and opinions |  |  |
| 9）Get access to useful knowledge and information |  |  |
| 10）Keep in line with people around who are using this SNS |  |  |
| 11）Give vent to emotions |  |  |
| 12）Get access to the latest news and popular topics |  |  |
| 13）Pass time |  |  |


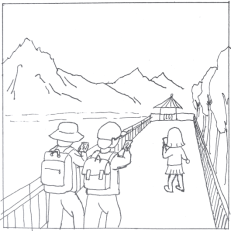


|  | YES | NO |
| --- | --- | --- |
| 1）Search for the latest information about others and know about friends’ recent status |  |  |
| 2）Entertain and recreate |  |  |
| 3）Record immediate ideas and feelings |  |  |
| 4）Follow the users, celebrities or organizations interested |  |  |
| 5）Keep up with trends and avoid being outdated |  |  |
| 6）Confide in others and get advice |  |  |
| 7）Rest and relax |  |  |
| 8）Express own attitudes and opinions |  |  |
| 9）Get access to useful knowledge and information |  |  |
| 10）Keep in line with people around who are using this SNS |  |  |
| 11）Give vent to emotions |  |  |
| 12）Get access to the latest news and popular topics |  |  |
| 13）Pass time |  |  |


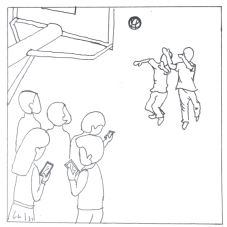


|  | YES | NO |
| --- | --- | --- |
| 1）Search for the latest information about others and know about friends’ recent status |  |  |
| 2）Entertain and recreate |  |  |
| 3）Record immediate ideas and feelings |  |  |
| 4）Follow the users, celebrities or organizations interested |  |  |
| 5）Keep up with trends and avoid being outdated |  |  |
| 6）Confide in others and get advice |  |  |
| 7）Rest and relax |  |  |
| 8）Express own attitudes and opinions |  |  |
| 9）Get access to useful knowledge and information |  |  |
| 10）Keep in line with people around who are using this SNS |  |  |
| 11）Give vent to emotions |  |  |
| 12）Get access to the latest news and popular topics |  |  |
| 13）Pass time |  |  |


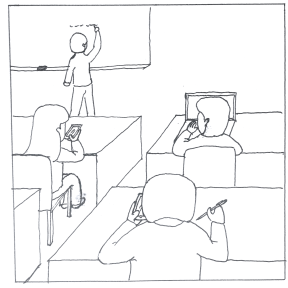


|  | YES | NO |
| --- | --- | --- |
| 1）Search for the latest information about others and know about friends’ recent status |  |  |
| 2）Entertain and recreate |  |  |
| 3）Record immediate ideas and feelings |  |  |
| 4）Follow the users, celebrities or organizations interested |  |  |
| 5）Keep up with trends and avoid being outdated |  |  |
| 6）Confide in others and get advice |  |  |
| 7）Rest and relax |  |  |
| 8）Express own attitudes and opinions |  |  |
| 9）Get access to useful knowledge and information |  |  |
| 10）Keep in line with people around who are using this SNS |  |  |
| 11）Give vent to emotions |  |  |
| 12）Get access to the latest news and popular topics |  |  |
| 13）Pass time |  |  |


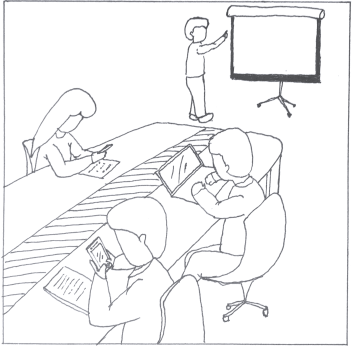


|  | YES | NO |
| --- | --- | --- |
| 1）Search for the latest information about others and know about friends’ recent status |  |  |
| 2）Entertain and recreate |  |  |
| 3）Record immediate ideas and feelings |  |  |
| 4）Follow the users, celebrities or organizations interested |  |  |
| 5）Keep up with trends and avoid being outdated |  |  |
| 6）Confide in others and get advice |  |  |
| 7）Rest and relax |  |  |
| 8）Express own attitudes and opinions |  |  |
| 9）Get access to useful knowledge and information |  |  |
| 10）Keep in line with people around who are using this SNS |  |  |
| 11）Give vent to emotions |  |  |
| 12）Get access to the latest news and popular topics |  |  |
| 13）Pass time |  |  |


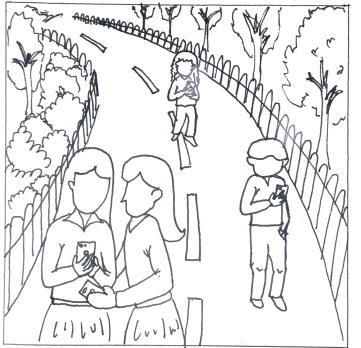


|  | YES | NO |
| --- | --- | --- |
| 1）Search for the latest information about others and know about friends’ recent status |  |  |
| 2）Entertain and recreate |  |  |
| 3）Record immediate ideas and feelings |  |  |
| 4）Follow the users, celebrities or organizations interested |  |  |
| 5）Keep up with trends and avoid being outdated |  |  |
| 6）Confide in others and get advice |  |  |
| 7）Rest and relax |  |  |
| 8）Express own attitudes and opinions |  |  |
| 9）Get access to useful knowledge and information |  |  |
| 10）Keep in line with people around who are using this SNS |  |  |
| 11）Give vent to emotions |  |  |
| 12）Get access to the latest news and popular topics |  |  |
| 13）Pass time |  |  |


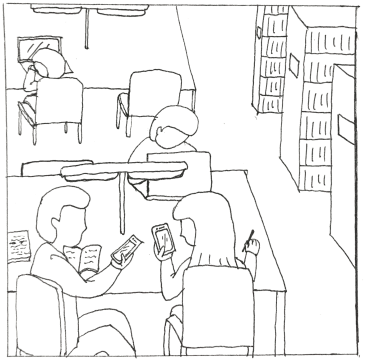


|  | YES | NO |
| --- | --- | --- |
| 1）Search for the latest information about others and know about friends’ recent status |  |  |
| 2）Entertain and recreate |  |  |
| 3）Record immediate ideas and feelings |  |  |
| 4）Follow the users, celebrities or organizations interested |  |  |
| 5）Keep up with trends and avoid being outdated |  |  |
| 6）Confide in others and get advice |  |  |
| 7）Rest and relax |  |  |
| 8）Express own attitudes and opinions |  |  |
| 9）Get access to useful knowledge and information |  |  |
| 10）Keep in line with people around who are using this SNS |  |  |
| 11）Give vent to emotions |  |  |
| 12）Get access to the latest news and popular topics |  |  |
| 13）Pass time |  |  |


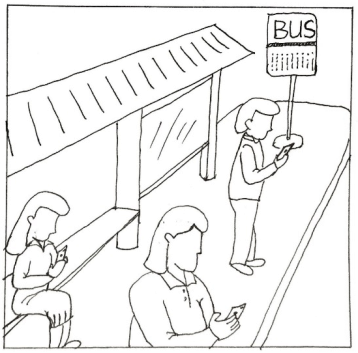


|  | YES | NO |
| --- | --- | --- |
| 1）Search for the latest information about others and know about friends’ recent status |  |  |
| 2）Entertain and recreate |  |  |
| 3）Record immediate ideas and feelings |  |  |
| 4）Follow the users, celebrities or organizations interested |  |  |
| 5）Keep up with trends and avoid being outdated |  |  |
| 6）Confide in others and get advice |  |  |
| 7）Rest and relax |  |  |
| 8）Express own attitudes and opinions |  |  |
| 9）Get access to useful knowledge and information |  |  |
| 10）Keep in line with people around who are using this SNS |  |  |
| 11）Give vent to emotions |  |  |
| 12）Get access to the latest news and popular topics |  |  |
| 13）Pass time |  |  |


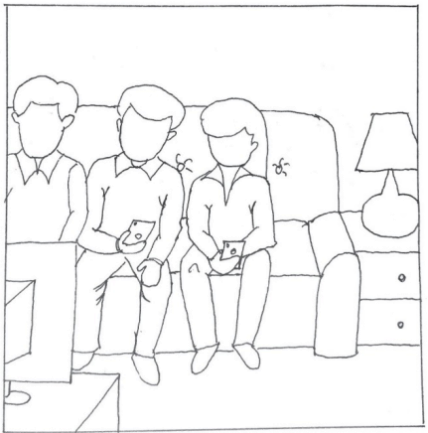


|  | YES | NO |
| --- | --- | --- |
| 1）Search for the latest information about others and know about friends’ recent status |  |  |
| 2）Entertain and recreate |  |  |
| 3）Record immediate ideas and feelings |  |  |
| 4）Follow the users, celebrities or organizations interested |  |  |
| 5）Keep up with trends and avoid being outdated |  |  |
| 6）Confide in others and get advice |  |  |
| 7）Rest and relax |  |  |
| 8）Express own attitudes and opinions |  |  |
| 9）Get access to useful knowledge and information |  |  |
| 10）Keep in line with people around who are using this SNS |  |  |
| 11）Give vent to emotions |  |  |
| 12）Get access to the latest news and popular topics |  |  |
| 13）Pass time |  |  |
